# Supplementary material for: Identification of photoperiod-induced specific miRNAs in the adrenal glands of Sunite sheep (Ovis aries)
Source: Front Vet Sci. 2022 Jul 22;9:888207. doi: 10.3389/fvets.2022.888207 (PMC9354845; doi:10.3389/fvets.2022.888207)
Supplement: Supplementary file 1 [file Data_Sheet_1.ZIP › Supplementary file/miRDeep2 file.zip!/miRDeep2 file/Know_Structure/SPLP42A1/oar-let-7i.pdf]

miRBase precursor : oar-let-7i
Total read count : 414553
oar-let-7i read count : 414379
remaining reads : 174

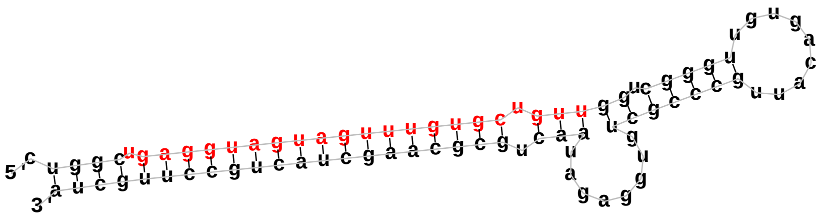

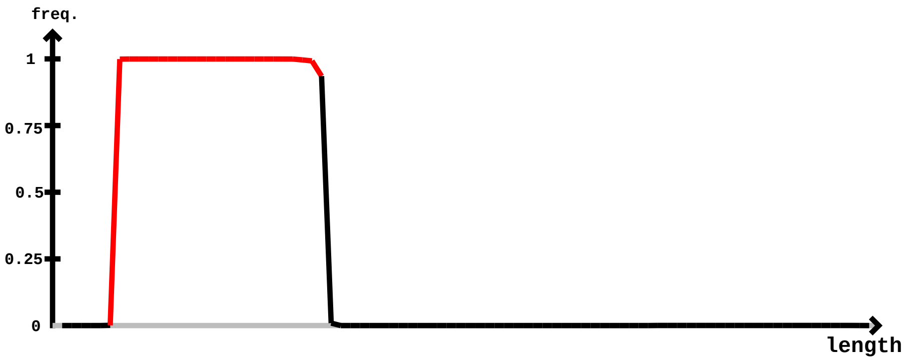

oar-let-7i

```
5'- cuggcugagguaguaguuugugcuguuggucggguugugacauugcccgcuguggagauaacugcgcaagcuacugccuugcua   -3'    exp
    .((((.(((((((((((((((((.(((((.(((((.........)))))))........))).)))))))))))))))))))))    reads      mm         sample
    ....cugagguaguaguuugugcu............................................................    1          0          seq
    ....cugagguaguaguuugugcug...........................................................    1          0          seq
    ....cugagguaguaguuugugcugu..........................................................    25         0          seq
    ....cugagguaguaguuugugcuguu.........................................................    352        0          seq
    ....cugagguaguaguuugugcuguug........................................................    5          0          seq
    .....ugagguaguaguuugug..............................................................    16         0          seq
    .....ugagguaguaguuugugc.............................................................    55         0          seq
    .....ugagguaguaguuugugG.............................................................    4          1          seq
    .....ugagguaguaguuugugA.............................................................    12         1          seq
    .....ugagguaguaguuugugcu............................................................    1323       0          seq
    .....ugagguaguaguuugugcug...........................................................    1552       0          seq
    .....ugagguaguaguuugugcugu..........................................................    23659      0          seq
    .....ugagguaguaguuugugcuguu.........................................................    383370     0          seq
    .....ugagguaguaguuugugcuguug........................................................    3803       0          seq
    .....ugagguaguaguuugugcuguugg.......................................................    12         0          seq
    .....ugagguaguaguuugugcuguuggu......................................................    2          0          seq
    .....ugagguaguaguuugugcuguuggucggguu................................................    1          0          seq
    ......gagguaguaguuugugcugu..........................................................    6          0          seq
    ......gagguaguaguuugugcuguu.........................................................    171        0          seq
    ......gagguaguaguuugugcuguug........................................................    4          0          seq
    .......agguaguaguuugugcuguu.........................................................    1          0          seq
    ..........uaguaguuugugcuguu.........................................................    5          0          seq
    .............................................................cugcgcaagcuacugccuu....    1          0          seq
    .............................................................cugcgcaagcuacugccuug...    3          0          seq
    .............................................................cugcgcaagcuacugccuugc..    73         0          seq
    .............................................................cugcgcaagcuacugccuugcu.    94         0          seq
    .............................................................cugcgcaagcuacugccuugcua    1          0          seq
    ..............................................................ugcgcaagcuacugccuugc..    1          0          seq
```
